# Clinical and analytical monitoring of patients with multiple sclerosis on anti-CD20 therapeutics: a real-world safety profile study

**DOI:** 10.3389/fneur.2024.1500763

**Published:** 2025-01-06

**Authors:** André Aires Fernandes, Ana Lídia Neves, Daniela Ferro, Mafalda Seabra, Teresa Mendonça, Ricardo Soares dos Reis, Maria José Sá, Joana Guimarães, Pedro Abreu

**Affiliations:** ^1^Unidade Local de Saúde de São João, Porto, Portugal; ^2^Department of Clinical Neurosciences and Mental Health, Faculty of Medicine, University of Porto, Porto, Portugal; ^3^Faculty of Health Sciences, Fernando Pessoa University, Porto, Portugal

**Keywords:** central nervous system, multiple sclerosis, anti-CD20 therapies, real-world data, hypogammaglobulinemia, lymphopenia

## Abstract

**Background:**

Anti-CD20 monoclonal antibodies are a class of immunosuppressive drugs widely used in the treatment of central nervous system (CNS) inflammatory diseases, with well-established efficacy and safety. Although rare, these therapies can be associated with serious adverse events including hematological and infectious complications. This study aims to evaluate their safety and tolerability profile in real-world clinical practice.

**Methods:**

We conducted a retrospective cohort study comprising patients diagnosed with multiple sclerosis (MS) treated with anti-CD20 drugs since 2016 followed in the Demyelinating Diseases clinic of a tertiary center. Clinical and paraclinical parameters were evaluated, including complete blood count and immunoglobulins measurements.

**Results:**

A total of 160 with multiple sclerosis (pwMS) were included in our study, of whom 110 (68.8%) were female and 147 are currently receiving anti-CD20 therapies. Half of the patients were diagnosed with relapsing–remitting MS, while the remaining had progressive forms, including 23 with primary-progressive MS and 57 with secondary-progressive MS. Eighty-three patients were on ocrelizumab, 48 on rituximab, and 29 on ofatumumab. The mean follow-up duration from the start of anti-CD20 therapy was 30.5 ± 21.3 months. During this period, serious adverse events were observed in 9 patients, including SARS-CoV-2 infection (resulting in one death), urinary tract infection, febrile neutropenia, severe diarrhea, and acute hepatitis. The rate of serious infections in the ocrelizumab subgroup was consistent with the literature, although a higher rate was observed in the rituximab subgroup. A positive correlation was found between serious infectious complications and lower IgG levels. Additionally, longer exposure to anti-CD20 therapy in our cohort was associated with an increased risk of IgG deficiency and a higher incidence of serious infections. Lymphopenia was detected in 25 patients, though it was not directly linked to the occurrence of serious infections.

**Conclusion:**

Our work confirms the tolerability and safety of anti-CD20 drugs in a real-world clinical practice MS cohort, despite their frequent association with analytical changes such as lymphopenia and hypogammaglobulinemia. To better understand the clinical significance of hypogammaglobulinemia secondary to anti-CD20 treatment and to develop strategies for mitigating the associated potential infection risk, future studies with larger populations are essential.

## 1 Introduction

Anti-CD20 monoclonal antibodies are a class of immunosuppressive drugs widely used in inflammatory diseases of the central nervous system (CNS), notably multiple sclerosis (MS) (1–4). These drugs selectively deplete CD20+ B and CD20+ T cells, which play an important role in multiple sclerosis (MS) pathogenesis. Currently, they are regarded as therapies effective for this disease, as they prevent relapses, reduce the number new or enlarging MS lesions and mitigate progression in relapsing–remitting MS (RRMS) (5).

Since the FDA approval of the chimeric monoclonal antibody rituximab in 1997, anti-CD20 therapy has become a cornerstone not only in the management of B-cell lymphoma but also in immune-mediated disorders (6). Indeed, rituximab was the first anti-CD20 therapeutic used in MS (5). HERMES and OLYMPUS, two randomized placebo-controlled phase 2 trials, have demonstrated the efficacy of rituximab as a disease-modifying therapy (DMT) for RRMS and primary progressive multiple sclerosis (PPMS), respectively (3, 7). Since there are no phase III clinical trials for rituximab in MS, the drug is not currently approved for this indication; however, it is frequently used off-label based on data from multiple observational studies that have demonstrated its efficacy and tolerability in MS (2). Ocrelizumab, was the first humanized anti-CD20 antibody approved by the Foods and Drug Administration (FDA) and the European Medicines Agency (EMA) for treating adults with RRMS with active disease or early PPMS with imaging features characteristic of inflammatory activity (8, 9). The OPERA I and II and the ORATORIO trials attested, respectively, its efficacy in relapsing forms of MS (RMS), including RRMS and secondary-progressive MS (SPMS) and in PPMS (8). In 2020, ofatumumab, was the first subcutaneous self-administered anti-CD20 humanized monoclonal antibody approved in RMS forms based on the results of the phase III ASCLEPIOS I and II studies (10). More recently, another intravenous anti-CD20 humanized monoclonal antibody, ublituximab, has shown efficacy in phase III clinical trials, ULTIMATE I and II, for RMS (11).

Despite sharing a similar mode of action, variations in pharmacological and clinical features have been observed among the mentioned therapies (5, 12). Particularly, differences in safety profiles, including administration-related reactions and other immune responses, exist among different antibodies (12). Infusion and injection-related reactions, along with respiratory and urinary tract infections, emerged as the most reported adverse events in people with MS (pwMS) participating in anti-CD20 MS clinical trials (5, 12). These distinct safety profiles should be carefully considered in conjunction with the needs of pwMS during treatment decision-making processes (12).

Randomized controlled trials (RCTs), although vital for determining the efficacy and safety of new drugs due their limited duration and strict inclusion criteria, may not capture long-term adverse effects of their sustained use in a real-life population of MS patients. This may be the case of the prolonged utilization of anti-CD20, which impairs many B cell immune functions, such as the immunoglobulin production (13, 14). Real-word studies may overcome this issue, since they provide valuable insights into treatment tolerability, effectiveness, and safety by examining outcomes in a broader and more representative pwMS population (13, 15). Given the recent use of anti-CD20 therapies in treating MS and other neuroinflammatory diseases, few studies have been published that address their tolerability and safety outcomes in a real-world cohort of pwMS (2, 15–18).

Our aim is to describe the tolerability and safety outcomes of anti-CD20 treatments on a real-world population of pwMS.

## 2 Materials and methods

### 2.1 Study design

We conducted a retrospective cohort study involving pwMS who were treated with anti-CD20 monoclonal antibodies between January 2016 and January 2024. People with MS were followed at a tertiary center outpatient demyelinating diseases clinic. The anti-CD20 antibody therapies included ofatumumab, ocrelizumab, and rituximab. MS diagnosis was established based on the revised 2017 McDonald criteria for MS, and the Lublin criteria definition was applied to classify the various MS phenotypes (RR, SP and PPMS) (19, 20).

### 2.2 Data collection

Baseline data, validated and collected from medical records, included: (a) demographic variables, (b) primary diagnosis, specifying the multiple sclerosis phenotype, (c) duration of MS at the initiation of anti-CD20 treatment, (d) previous disease-modifying therapy before starting anti-CD20 drug, (e) neurological assessment, including baseline Expanded Disability Status Scale (EDSS) scores, and (f) details regarding the type and administration regimen of the anti-CD20 drug therapy.

Paraclinical parameters were collected at various intervals, preferably on the day of each anti-CD20 dose administration or during neurology appointments, both prior to initiating the drug and every four or 6 months thereafter. These included a complete blood count, liver function tests [comprising alanine transaminase (ALT), aspartate transaminase (AST), alkaline phosphatase (ALP), gamma-glutamyl transferase (GGT), and serum bilirubin], and immunoglobulin measurements. Patient follow-up occurred every 6 months. Lymphopenia severity, when present, was graded using the National Cancer Institute’s Common Terminology Criteria for Adverse Events: grade 1 [absolute lymphocyte count (ALC) 800–1,000 lymphocytes/μl], grade 2 (ALC 500–799 lymphocytes/μl), grade 3 (ALC 200–499 lymphocytes/μl), and grade 4 (ALC <200/μl) (21).

Immunoglobulin levels were assessed against age-specific and laboratory-specific lower limit of normal (LLN) values, with hypogammaglobulinemia defined as IgG < 650 mg/dL and IgM <55 mg/dL. Also, all patients were tested for globulin levels prior to initiation of anti-CD20 therapy.

Adverse events (AEs) information was gathered from hospital electronic medical records, including reviews of emergency episodes. Additionally, adverse clinical effects were assessed during periodic follow-up consultations, typically conducted every 4–6 months annually, or sooner in case of any unexpected clinical alterations. AEs were then registered and graded according to the National Cancer Institute’s Common Terminology Criteria for Adverse Events (15). We also classified AEs accordingly to their temporal profile: (a) immediate or during infusion/injection reactions AEs as those occurring during or within 24 h of any anti-CD20 administration; (b) peri-infusion/injection adverse events occurring within the first 7 days after anti-CD20 administration; and (c) post-infusion/injection as those occurring months to years’ after anti-CD20 administration including infections and cardiovascular, cerebral, gastrointestinal, and pulmonary complications (22, 23).

Uncomplicated upper respiratory tract and lower urinary tract infections were excluded from analysis due to potential underreporting. Patient discontinuation of anti-CD20 therapy and reasons for discontinuation were also documented from clinical records.

### 2.3 Statistical analyses

Statistical analysis was performed using SPSS version 27. Clinical and paraclinical characteristics were presented as mean ± standard deviation (SD). Normality of data was assessed using the Kolmogorov–Smirnov test. Group comparisons were made using the *χ*^2^ test, Mann–Whitney *U* test, and Student’s *t*-test, as appropriate (*p*-values <0.05 were deemed statistically significant).

This study was conducted according to the principles of the Helsinki declaration and received approval from the local ethics committee.

## 3 Results

### 3.1 Study population and therapeutic characterization

A total of 160 pwMS were enrolled, with 110 (68.8%) being female. The demographic and clinical data are summarized in Table 1. Among our cohort, 80 (50%) participants exhibited a relapsing–remitting disease course, while the remaining presented with progressive forms, including 23 (14.4%) individuals with primary-progressive and 57 (35.6%) with secondary-progressive MS. Ocrelizumab was the most frequently prescribed anti-CD20 drug (*n* = 83, 51.9%), followed by rituximab (*n* = 48, 30%) and ofatumumab (*n* = 29, 18.1%). The mean follow-up time, measured from the initiation of anti-CD20 therapy, was 30.3 ± 21.5 months, with the rituximab subgroup having the longest exposure duration at 50.6 ± 24.0 months. In our cohort, 45 (28.1%) pwMS were treatment-naïve and 115 (71.9%) pwMS were exposed to at least one DMT previously (Table 2). Therapies prior to anti-CD20 initiation included (*n* = 115): fingolimod (*n* = 26, 22.6%), beta-interferon (*n* = 22, 19.1%), dimethyl fumarate (*n* = 18, 15.7%), glatiramer acetate (*n* = 13, 11.3%), natalizumab (*n* = 13, 11.3%), teriflunomide (*n* = 8, 7.0%), cladribine (*n* = 6, 5.2%), rituximab (*n* = 4, 3.5%), intravenous human immunoglobulin (*n* = 2, 1.7%), methotrexate (*n* = 1, 0.87%), and human autologous stem cell transplant (*n* = 1, 0.87%).

**Table 1:** Demographic and clinical data.

Gender (female)	113 (68.9%)
Mean age (last evaluation)	49.4 ± 12.0
Anti-CD20 therapy	
Ocrelizumab	83 (51.9%)
Rituximab	48 (30.0%)
Ofatumumab	29 (18.1%)
MS phenotype	
RRMS	80 (50.0%)
SPMS	57 (35.6%)
PPMS	23 (14.4%)
Overall EDSS score (median, range)	
Prior to anti-CD20 initiation	3.5 (0–8.0)
At last evaluation	4.0 (0–8.0)
Time of exposure (months)	30.5 ± 21.3
Rituximab	50.6 ± 24.0
Ocrelizumab	25.7 ± 12.6
Ofatumumab	10.5 ± 5.6

CNS, central nervous system; EDSS, Expanded Disability Status Scale; PPMS, primary-progressive multiple sclerosis; RRMS, relapsing–remitting multiple sclerosis; SPMS, secondary-progressive multiple sclerosis.

**Table 2:** Previous therapeutic regimens.

Previous therapeutic regimens	N
Treatment-naïve	48
Fingolimod	26
Beta-interferon	22
Dimethyl fumarate	18
Glatiramer acetate	13
Natalizumab	13
Teriflunomide	8
Cladribine	6
Rituximab	4
Intravenous immunoglobulin	2
Methotrexate	1
Human autologous stem cell transplant	1

### 3.2 Paraclinical findings and adverse events

The main paraclinical alterations and adverse effects are described in Tables 3–5, respectively, and the reasons for anti-CD20 therapy discontinuation are depicted in Table 6.

**Table 3:** Anti-CD20 induced lymphopenia (grade 1: 800–1,000 lymphocytes/μl, grade 2: 500–799 lymphocytes/μl, grade 3: 200–499 lymphocytes/μl).

	Lymphopenia (*n*)			
Anti-CD20	Grade 1	Grade 2	Grade 3	Total
Ocrelizumab	6	5	1	12
Rituximab	2	4	2	8
Ofatumumab	5	0	0	5

**Table 4:** Anti-CD20 induced Hypogammaglobulinemia.

	Hypogammaglobulinemia (*n*)		
Anti-CD20	Low IgM	Low IgG	Not available
Ocrelizumab	30	9	26
Rituximab	20	6	13
Ofatumumab	4	0	17
Total	54	15	56

**Table 5:** Reported adverse events, graded according to the National Cancer Institute’s Common Terminology Criteria for Adverse Events (21).

Patient	MS course	EDSS	Anti-CD20 drug	Exposure time (in months)	Previous DMT	Adverse events	Adverse events classification	Management
F, 51yo	PP	6.0	Ocrelizumab	16	Rituximab	Myalgia	2	DMT switch
F, 64yo	RR	6.5	Ocrelizumab	46	Fingolimod	Flu-like syndrome	2	Favorable outcome with symptomatic care
F, 27yo	RR	0	Ocrelizumab	9	Treatment-naïve	Flu-like syndrome	2	Favorable outcome with symptomatic care
M, 46yo	RR	5.5	Ocrelizumab	31	Fingolimod	Flu-like syndrome	2	Favorable outcome with symptomatic care
F, 49yo	PP	3.5	Ocrelizumab	50	Glatiramer acetate	Diarrhea	2	Self-limiting event, spontaneous remission
F, 68yo	PP	7.0	Ocrelizumab	32	Treatment-naïve	Skin infection	2	Self-limiting event, spontaneous remission
F, 48yo	RR	4.0	Ocrelizumab	34	Fingolimod	Dental infection	2	Self-limiting event, spontaneous remission
F, 57yo	PP	5.5	Ocrelizumab	39	Treatment-naïve	Urinary tract infection	2	Favorable outcome with oral antibiotic
F, 36yo	RR	1.5	Ocrelizumab	6	Treatment-naïve	Crohn disease	3	DMT switch
M, 25yo	SP	2.0	Ocrelizumab	26	Natalizumab	Febrile neutropenia	4	Favorable outcome with filgrastim and broad-spectrum antibiotics → DMT switch
M, 58yo	PP	6.5	Ocrelizumab	6	Treatment-naïve	SARS-CoV-2 infection resulting in death	5	–
F, 73yo	PP	6.5	Rituximab	25	Treatment-naïve	SARS-CoV-2 infection	2	Favorable outcome with symptomatic care
M, 54yo	SP	6.0	Rituximab	41	Natalizumab	SARS-CoV-2 infection	2	Favorable outcome with symptomatic care
M, 62yo	SP	6.0	Rituximab	71	Glatiramer acetate	SARS-CoV-2 infection	2	Favorable outcome with symptomatic care
F, 60yo	SP	7.0	Rituximab	80	Dimethyl fumarate	Urinary tract infection	2	Favorable outcome with oral antibiotic
M,74yo	SP	7.5	Rituximab	35	Intravenous immunoglobulin	SARS-CoV-2 infection	3	Hospitalization with favorable outcome
F, 45yo	SP	6.5	Rituximab	86	Treatment-naïve	SARS-CoV-2 infection	3	Hospitalization with favorable outcome
F, 51yo	SP	6.5	Rituximab	70	Dimethyl fumarate	SARS-CoV-2 infection	3	Hospitalization with favorable outcome
M, 65yo	SP	3.5	Rituximab	34	Treatment-naïve	SARS-CoV-2 infection	3	Hospitalization with favorable outcome
F, 64yo	SP	7.5	Rituximab	76	Glatiramer acetate	Urinary tract sepsis	4	Hospitalization with favorable outcome
F, 51yo	SP	6.5	Rituximab	63	Dimethyl fumarate	SARS-CoV-2 infection and urinary tract infection	4	Hospitalization with favorable outcome
M, 51yo	RR	3.5	Ofatumumab	14	Fingolimod	Urinary tract infection	2	Favorable outcome with oral antibiotic
M, 37yo	RR	2.5	Ofatumumab	7	Fingolimod	Superior respiratory tract infection	2	Favorable outcome with oral antibiotic
M, 36yo	RR	1.0	Ofatumumab	14	Treatment-naïve	Diarrhea	2	Self-limiting event, spontaneous remission

DMT, disease-modifying therapy; M, male; MS, multiple sclerosis; NA, not applicable; F, female; PP, primary progressive; SP, secondary progressive, RR, relapsing–remitting; yo, years-old.

**Table 6:** Reasons for anti-CD20 therapy discontinuation.

Previous therapeutic regimens	Anti-CD20 (*n*)	
	Ocrelizumab	Rituximab
Therapeutic failure	1	8
Patient intolerance	1	
Adverse events		
Myalgia	1	
Febrile neutropenia	1	
Persistent grade 2 lymphopenia		1

#### 3.2.1 Paraclinical alterations

During the follow-up period, the main paraclinical alterations found consisted of lymphopenia, Hypogammaglobulinemia and a single case of severe neutropenia. In our cohort, lymphopenia was detected in 25 patients (15.6%), with 13 (52.0%) corresponding to grade 1 (800–1,000 lymphocytes/μl), 9 (36.0%) to grade 2 (500–799 lymphocytes/μl), and 3 (12.0%) to grade 3 (200–499 lymphocytes/μl) (Table 3). Lymphopenia frequency did not significantly differ among the three anti-CD20 drugs. Its onset may occur early, at 6 months or up to 33 months after drug initiation, with a mean time of 7.6 months.After adjusting for the anti-CD20 drug, age, time of exposure, and EDSS, we observed that men have a significantly higher risk (OR 3.027, *p* < 0.05) of developing lymphopenia, without additional associations found (Table 7). Lymphopenia was not associated with infectious adverse events (Table 8). Apart from lymphopenia, we also registered a single case of febrile neutropenia that developed following ocrelizumab infusion, resulting in hospitalization due to sepsis with an unidentified focus.

**Table 7:** Associations found between different sociodemographic/clinical features and lymphopenia, accessed with Chi-square test and Mann–Whitney *U* test or Student’s *t*-test (based on normality tests), for categoric and continuous variables, respectively.

	*p*
Age	0.807
Gender (male)	0.014*
EDSS	0.257
Time of exposure	0.963

* After adjusting for the anti-CD20 drug, age (actual), time of exposure and EDSS score using a binary logistic regression model, it was observed that men have a significantly higher risk, (OR 3.017, *p* < 0.05), of developing lymphopenia.

We detected hypogammaglobulinemia in approximately one third of our cohort, with 54 patients (33.8%) having IgM values under the LLN (median: 31.3 mg/dL; range: 4–54 mg/dL, reference value ≥55 mg/dL) and 15 patients (9.4%) having low IgG values (median: 582.9 mg/dL; range: 512–643 mg/dL, reference value ≥650 mg/dL). We observed that hypogammaglobulinemia may manifest either early, during the initial laboratory evaluation at six-months of treatment, or later, up to 54-months after drug initiation (mean of 14.0 ± 11.3 months). Hypogammaglobulinemia did not statistically differ between ocrelizumab and rituximab; however, it was less frequent in the subgroup on ofatumumab (*p* < 0.01). Age or EDSS score were not independently associated with the risk of developing hypogammaglobulinemia (*p* ≥ 0.05) (Table 9). A positive correlation was observed between the occurrence of serious infections and lower IgG levels (*p* = 0.01). Longer exposure time was found to predispose to lower IgG levels (*p* = 0.02) and to the occurrence of serious infections (*p* = 0.02) (Tables 8, 9). Hypogammaglobulinemia did not lead to anti-CD20 discontinuation. Persistent hypogammaglobulinemia was identified in 36 patients (22.5%), primarily affecting only IgM levels. Among these, 24 patients (66.7%) were on ocrelizumab, 10 patients (28.8%) were on rituximab, and two patients (5.6%) were on ofatumumab.

**Table 8:** Anti-CD20 dependent factors and risk of serious infections, accessed with Chi-square test and Mann–Whitney *U* test or Student’s *t*-test (based on normality tests), for categoric and continuous variables, respectively.

	*p*
Lymphopenia	0.454
Low IgG levels	0.011
Low IgM levels	0.273
Time of exposure	0.022
EDSS	0.023

**Table 9:** Associations found between different sociodemographic/clinical features and hypogammaglobulinemia, accessed with Chi-square test and Mann–Whitney *U* test or Student’s *t*-test (based on normality tests), for categoric and continuous variables, respectively.

	*p*	
	IgM	IgG
Age	0.689	0.545
Gender	0.665	0.576
EDSS	0.365	0.253
Time of exposure	0.194	0.024

#### 3.2.2 Adverse effects according to temporal profile

##### 3.2.2.1 Immediate infusion/injection reactions

A single infusion reaction characterized by diaphoresis was observed in one patient receiving rituximab therapy. No adverse effects related to subcutaneous injection (ofatumumab) were recorded.

##### 3.2.2.2 Peri-infusion adverse events

No peri-infusion AEs (within the first 7 days) were reported following the infusion, including infections.

##### 3.2.2.3 Post-infusion adverse events

Post-infusion AEs were reported in 24 patients (15.0%), with 11 patients treated with ocrelizumab, 10 with rituximab, and three with ofatumumab (Table 5). Post-infusion non-infectious AEs were observed in 7 (4.4%), mainly affecting the ocrelizumab subgroup (*n* = 6), consisting of flu-like syndrome in two patients, two cases of diarrhea, one of whom was subsequently diagnosed with Crohn’s disease, and myalgia in one patient. One patient under ofatumumab reported diarrhea after infusion. Post-administration infectious AEs were seen in 17 (10.6%) patients and were more frequent in the rituximab group (*n* = 10, 58.8%, *p* = 0.042). Serious infections (grades 4 and 5) were observed in eight (5.0%) patients, including six patients treated with rituximab and two with ocrelizumab (*p* = 0.020). EDSS score was independently associated with the risk of serious infections (*p* = 0.023). Serious SARS-CoV-2 infections were more frequent in the rituximab subgroup (*n* = 6, *p* = 0.014), followed by ocrelizumab (*n* = 2). One fatality was reported due to SARS-CoV-2 infection in a patient under ocrelizumab after four infusions. As already mentioned, a single case of febrile neutropenia was observed 6 weeks following the second ocrelizumab infusion, resulting in hospitalization due to sepsis with an unidentified focus. Despite the severity of this condition, the patient had a favorable outcome following treatment with recombinant human granulocyte colony-stimulating factor (filgrastim) and broad-spectrum antibiotics. Nevertheless, a new demyelinating lesion was observed 6 months after this event, leading to the decision to switch to ofatumumab. The remaining hospitalizations were attributed to a urinary tract infection in one patient receiving rituximab and a single case of severe diarrhea associated with a recent diagnosis of Crohn’s disease, occurring 5 months after the last infusion of ocrelizumab (the patient is currently on natalizumab). No cases of progressive multifocal leukoencephalopathy or malignancy were identified in our cohort.

### 3.3 Anti-CD20 discontinuation

Within the initial cohort of 160 patients, 13 (8.1%) patients underwent discontinuation of anti-CD20 therapy. This therapeutic interruption predominantly implicated two specific agents: rituximab (*n* = 9, 69.2%) and ocrelizumab (*n* = 4, 30.7%). Reasons for discontinuation included therapeutic failure in nine patients (rituximab = 8, ocrelizumab = 1), occurrence of adverse events and/or patient intolerance in four patients, including single cases of myalgia and febrile neutropenia under ocrelizumab and a persistent grade 2 lymphopenia in patient treated with rituximab (table 6). The median duration of therapy prior to discontinuation was 26 months.

## 4 Discussion

In our real-world study, we evaluated a cohort of MS patients treated with anti-CD20 therapies since 2016. The anti-CD20 therapies used were rituximab, ocrelizumab, and more recently ofatumumab. According to our results, rituximab seems to be associated with the highest risk of infectious AE including serious infections among the anti-CD20 therapies. We did not find significant differences among anti-CD20 drugs regarding laboratory parameters. In this study, IgG deficiency and exposure time seem to predispose to a higher risk of serious infection. Surprisingly, we found that men may have a superior risk of developing lymphopenia. However, lymphopenia was not associated with an increased risk of serious infectious events. Indeed, the role of lymphopenia in the risk of serious infections in anti-CD20 therapies remains a subject of debate. In a cohort of patients with demyelinating diseases receiving anti-CD20 therapies, Oksbjerg et al. (1) and Mears et al. (24) reported a positive association between lymphopenia and the risk of severe infection. However, this finding has not been consistently reproduced in subsequent cohorts, raising questions about the true impact of lymphopenia on infection risk (25). As observed in our cohort, lymphopenia secondary to anti-CD20 therapy tends to be mild, predominantly grade 1 or 2, with severe lymphopenia (grade 3 or higher) observed in less than 2% of cases (1, 24, 25). This, combined with a low incidence of serious infectious events (5%), may partially explain the lack of association between lymphopenia and the risk of severe infection. However, the clinical relevance of severe lymphopenia remains unclear and warrants further investigation. In our work the percentage of patients with lymphopenia was 13.3% of ocrelizumab-treated patients compared to 20.7% reported in the clinical trials (7, 26). In the subgroup treated with rituximab, this percentage was higher (17.3%), perhaps reflecting a longer median exposure time (50.6 months versus 25.7 months). Lymphopenia is, somewhat, an expected outcome since these treatments efficiently deplete CD20+ B cells, as well as decrease T cell populations (27).

A single case of febrile neutropenia was observed in a patient undergoing treatment with ocrelizumab. Despite the absence of the CD20 receptor on neutrophils, neutropenia remains an uncommon but important adverse event associated with both rituximab and ocrelizumab, with incidence rates reported at 4.5–6.5% for rituximab and 1% for ocrelizumab, respectively (28, 29). Typically, neutropenia associated to anti-CD20 manifests with a delayed onset, generally occurring more than 4 weeks after the last infusion, however, in our patient, it developed merely 10 days following their last ocrelizumab infusion (26, 30). Although the exact mechanism of neutropenia induced by anti-CD20 remains unknown, several theories such as immunomediated mechanisms, silent infection, or neutrophil apoptosis triggered by the FAS/FAS ligand pathway have been proposed (23).

Hypogammaglobulinemia was the most frequent laboratorial abnormality observed in our cohort. Although the underlying mechanism for the development of hypogammaglobulinemia is, until now, unknown, diminished levels of B-cell-secreted cytokines, such as BAFF and interleukin 6, may determine a reduction in the formation of plasma cells from precursors and therefore diminish immunoglobulins secretion (9, 23). The protocols of the ocrelizumab OPERA I/II and ORATORIO trials excluded patients with pre-randomization IgG levels 18% below LLN (<460 mg/dL) or IgM levels 8% below LLN, which limited the trials’ ability to capture drug-related risk of hypogammaglobulinemia. Nevertheless, there is consistent evidence from clinical trials and real-word data, that hypogammaglobulinemia is perhaps the most expected laboratory abnormality in patients treated with anti-CD20 drugs (31). Indeed, real-world data from the Danish MS registry showed that the prevalence of low IgM and IgG levels among patients treated with CD20-depleting therapies (including rituximab, ocrelizumab, and ofatumumab) were 28 and 5%, respectively (1). In our cohort, superior rates were observed, 33.8 and 9.4%, respectively, and no statistically significant differences were observed among the subgroups receiving rituximab and ocrelizumab regarding this laboratory alteration. When looking at the different immunoglobulins, in our cohort, IgM was the most frequently affected immunoglobulin in the ocrelizumab and rituximab groups, confirming what is described in other studies (2, 6, 28). Only four patients (13.8%, *n* = 4) treated with ofatumumab developed hypogammaglobulinemia affecting IgM (13.8%, *n* = 4), however the short follow up time in this subgroup and the high percentage of data not available (*n* = 17, 58.6%) must be considered. Furthermore, clinical trial evidence suggests that not all anti-CD20 therapies have the same impact on IgG levels. For example, the ofatumumab clinical trial program did not observe decreases in IgG levels (10). Although the exact reasons remain unclear, it has been proposed that variations in the mechanisms of B cell depletion may account for the differing hypogammaglobulinemia profiles among anti-CD20 therapies. These differences may be related to the distinct epitopes targeted and the biochemical structures of the antibodies (32). Another possible explanation is that subcutaneously administered antibodies, such as ofatumumab, do not induce the same level of B cell depletion in the spleen as intravenously administered antibodies (33, 34). In the present study, we found lower IgG levels in patients with infections requiring hospitalization (*p* = 0.011). Indeed, according to the literature, low IgG appear to be correlated with the risk of infection, whereas no such association is observed with IgM levels (35). Currently, there are no consensus guidelines for monitoring serum immunoglobulin levels or managing the risk of hypogammaglobulinemia in patients with MS (31). A multidisciplinary approach to care is critical to shift the focus from merely managing infectious complications to implementing a comprehensive system of assessment (beginning at MS diagnosis) and prevention strategies, including vaccination and prophylaxis (31, 36, 37). Modifications to treatment regimens, including dose reduction or extension of dosing intervals, are not currently advised due to the lack of sufficiently consistent and robust scientific evidence to support their efficacy (31, 38–41). In patients with hypogammaglobulinemia but without severe or recurrent infections, active surveillance for infectious complications, monitoring of immunoglobulin levels every 6 months, and reassessment of disease-modifying therapy based on clinical and laboratory risk factors are recommended (31). In the absence of well-established guidelines pertaining this issue, the patients identified with hypogammaglobulinemia have not undergone immunoglobulin replacement therapy, since they, until now did not had had serious or recurrent infections.

As in the clinical trials and observational studies of anti-CD20 drugs, the most common infectious AE observed in our study were respiratory and urinary tract infections (2, 7, 8, 10, 28). Contrary to previous data, the rituximab group exhibited a higher rate of infection-related AE compared to the ocrelizumab group (20.8% versus 15.2%) (42).

In patients treated with rituximab, the incidence of serious infections was 12.5% (*n* = 6), a rate higher than that reported in previous observational studies (1.7%) and in the two randomized, placebo-controlled phase 2 trials, HERMES (2.9%) and OLYMPUS (4.5%) (3, 7, 16). A plausible explanation for this discrepancy is that, compared to those studies, our cohort had a higher mean age (56.6 years) and a higher median EDSS (6.0) prior to rituximab initiation, indicating a, perhaps, more vulnerable patient population (3, 7, 16).

We observed two serious infections (grades 4 and 5) in the ocrelizumab-treated subgroup (2.4%), compared to rates of 1.3% in the OPERA trials and 6.2% in the ORATORIO trial, with observational studies reporting rates ranging from 0 to 7% (8, 15, 17, 18). Overall, the observed mortality rate in the ocrelizumab subgroup (1.3%) was slightly higher than that reported in the ORATORIO (0.8%) and OPERA II (0.2%) trials. In our study, during the follow-up period, one fatality was recorded in a patient receiving ocrelizumab, attributed to COVID-19. This person was older (58 years) and significantly disabled. We hypothesize that ocrelizumab may have contributed to a reduced immune response in an already more fragile patient.

Discontinuation of anti-CD20 therapy was observed in 13 patients (8.1%). Of these, nine patients discontinued due to therapeutic failure, while only four patients (ocrelizumab = 3, rituximab = 1) discontinued due to adverse events and/or patient intolerance. This low rate of therapy discontinuation is consistent with previous findings that demonstrate the tolerability and efficacy of anti-CD20 therapies (2, 7, 10, 25, 34).

We acknowledge several limitations in our study. The retrospective design inherently carries certain drawbacks, particularly the reliance on the quality and completeness of clinical records. Concomitant medical comorbidities, such obesity and diabetes mellitus, were not evaluated, which may modulate the risk of infectious AE. Clinical information on mild infusion reactions and mild infections may be underreported. For example, mild urinary tract infections might have been overlooked if managed by general practitioners, resulting in incomplete data. Although all patients underwent a complete blood count prior to rituximab or ocrelizumab infusions, or ofatumumab administration, immunoglobulin measurement is not standardized at our center. In fact, some patients had only one measurement annually, and it was not performed in 56 patients (34.1%), with the ofatumumab subgroup being disproportionately affected (*n* = 17, 65.4%). Also, ofatumumab was only approved in our center in 2022, which accounts for the reduced number of patients and the short follow up time in this subgroup, which can subsequently affect the rate of occurrence of analytical and clinical adverse events. Additionally, the impact of COVID19 vaccination was not able to be evaluated since the vaccination status was not evaluated.

In conclusion, anti-CD20 monoclonal antibodies have significantly impacted the treatment of multiple sclerosis, with their efficacy demonstrated in both randomized clinical trials and observational studies. However, these therapies are often associated with analytical changes, such as lymphopenia and hypogammaglobulinemia, which necessitate close monitoring. Although serious clinical-analytical adverse events are rare, the widespread and prolonged use of these drugs may increase their occurrence, as observed in our cohort. Our findings underscore the importance of regular clinical and analytical follow-up, including complete blood counts and immunoglobulin measurements. Currently, there are no consensus guidelines for monitoring serum immunoglobulins or managing the risk of hypogammaglobulinemia in MS patients. Future studies with larger cohorts are essential to fully understand the clinical implications of hypogammaglobulinemia secondary to anti-CD20 therapies and to develop strategies for mitigating the potential infection risk. Nonetheless, our study confirms the tolerability and safety of anti-CD20 drugs in real-world clinical practice for patients with neuroinflammatory diseases of the CNS, particularly multiple sclerosis.

## Data Availability

The raw data supporting the conclusions of this article will be made available by the authors, without undue reservation.
